# Longevity‐CancerDB: unlocking the distinctive features and roles of longevity‐associated genes in tumourigenesis

**DOI:** 10.1002/ctm2.1557

**Published:** 2024-01-22

**Authors:** Li Guo, Daoliang Xia, Jiaming Jin, Shizheng Xiong, Xinru Xu, Lulu Luo, Xueni Yang, Xinmiao Zhao, Dekang Ren, Jiafeng Yu, Tingming Liang

**Affiliations:** ^1^ State Key Laboratory of Organic Electronics and Information Displays Institute of Advanced Materials (IAM) Nanjing University of Posts and Telecommunications Nanjing China; ^2^ Jiangsu Key Laboratory for Molecular and Medical Biotechnology School of Life Science Nanjing Normal University Nanjing China; ^3^ Shandong Provincial Key Laboratory of Biophysics Institute of Biophysics Dezhou University Dezhou China

Dear Editor,

1

To explore relationship between longevity and cancer biology, a total of 400 screened longevity‐related genes were performed multi‐omics analysis to understand molecular features and validate prognostic values in aging‐related tumourigenesis. Further, we developed Longevity‐CancerDB (http://tmliang.cn/longene/), a user‐friendly web‐based resource, to provide the detailed information and pan‐cancer analysis using online analysis tools, which would contribute to exploring the molecular characteristics and clinical application of longevity‐associated genes in cancer.

Based on the worldwide aging, the genetics of human longevity have been widely concerned. A series of longevity‐associated genes have been reported, which may be potential targets for the drug development.^1^ Some genes may suppress tumourigenesis, such as FoxO3, a pro‐longevity gene, a target of p53.^2^ Some paradoxes based on the study of regulation of longevity are frequently linked to cancer, but longevity‐associated genes may also have important roles in aging‐related tumourigenesis. The heritability of longevity still remains unclear in part because of the complexity of the phenotypic trait,^3^ and the detailed relationship between the tumourigenesis and longevity is still far from clear, although these may provide specific data reference for unrevealing the cancer pathophysiology. As aging‐related diseases, it is critical to develop therapeutic approaches to balance longevity, tumour suppression, and cancer.^4^

Herein, a total of 400 longevity‐associated genes were screened from published literatures (Table S1), including homologous members in the gene family, such as Sirtuin and FOXO gene families. About 9.50% genes were located on Chr6, followed by Chr1 and Chr11 (Figure 1A). Many genes were prone to be characterised as cancer‐related genes with a role in multiple hallmarks of cancer and biological processes (Figures 1B–E and S1A,B), and the most enriched pathways were mainly focus on cancer‐related pathways, followed by insulin and mTOR signalling pathways that were longevity‐related pathways (Figure 1E–G). The gene set showed the significant expression and functional difference in cancer (Figure 1G,H), which might contribute to revealing the potential roles and application in cancer diagnosis and therapy. Some longevity‐related genes were potential drug targets, particularly in epidermal growth factor receptor (EGFR), IGF1R, and PI3K/MTOR signalling pathways (Figure 2A), implicating their critical roles in the cancer treatment. EGFR, the epidermal growth factor receptor, is involved in multiple biological processes, such as cell proliferation, apoptosis, invasions and metastasis, and it has been proposed for the cancer therapy.^5^, ^6^ Some genes were involved in changes at different molecular levels, such as abnormal expression patterns (Figures 2B and S1C–F) and higher mutation frequencies (Figure S2A–D) in cancer. Further analysis showed difference in overall survival between groups with different expressions (Figure 2D–E), indicating the potential correlation with cancer prognosis. Higher mutation frequencies may be associated with cancer outcomes, such as TP53, a key tumour suppressor gene (TSG).^7^, ^8^ Genes with higher mutation frequencies were mainly involved in PI3K, TP53 and cell cycle pathways (Figure S3A–C), and the mutations may be associated with prognosis (Figure S3D–F). Furthermore, based on the potential function of long genes in tumourigenesis, we found that longevity‐associated genes tended to be long genes (possessed 45.25%) (Figure S4A), and diverged length distributions also showed inconsistent expression patterns in different groups according to clinical features (Figure S4B–F).

**FIGURE 1:**
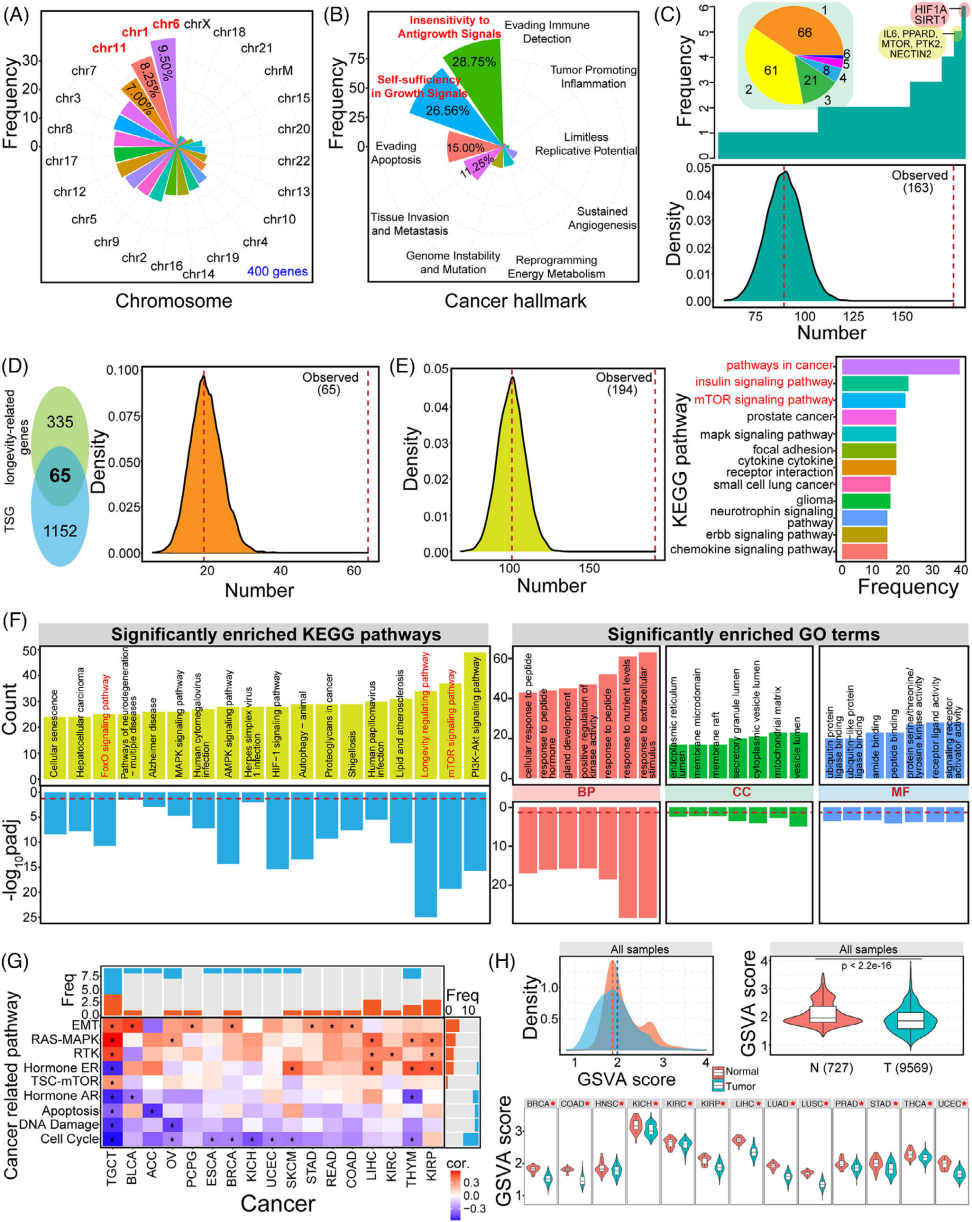
The potential roles of longevity‐associated genes in cancer. (A) The detailed distribution of the screened 400 longevity‐associated genes on human chromosomes (chr). Chromosomes 6 (9.50%), 1 (8.25%), and 11 (7.00%) are the dominant chromosomes containing more genes than other chromosomes (the percentages are also presented). (B) A total of 163 longevity‐associated genes (40.75%) are characterised as members in hallmarks of cancer, especially in insensitivity to antigrowth signals and self‐sufficiency in growth signals (some genes may be simultaneously detected in multiple hallmarks). (C) The detailed frequency distribution for genes that are identified as members in hallmarks of cancer, and several genes are highlighted because they are simultaneously detected in multiple hallmarks. The pie distribution shows the frequencies of genes as members of different hallmarks, indicating that these genes have important roles in tumourigenesis. To verify the result, randomly selected 400 genes from all protein‐coding genes (20 017 protein‐coding genes according to gencode.v41.annotation.gtf) are performed distribution analysis to assess whether the result is random (repeating 10 000 times). The below panel indicates that fewer genes in the randomly selecting gene set (*n* = 400) are detected roles in cancer hallmarks than longevity‐associated genes. The median value of 10 000 values based on random sampling is 86, and the observed value of longevity‐associated genes is 163 that is significantly higher than that in the randomly selected gene set, indicating that longevity‐associated genes tend to be genes with an important role in hallmarks of cancer. (D) The common 65 genes can be detected between longevity‐associated genes and tumour suppressor genes (TSG) obtained in OncoKB. The right panel indicates that fewer genes in the randomly selecting gene set (*n* = 400) are detected as TSG than longevity‐associated genes (repeating 10 000 times). The median value of 10 000 detected values is 19, and the observed value based on longevity‐associated genes is 65 that is significantly higher than that in randomly selected gene set, indicating that longevity‐associated genes tend to be TSG. (E) The left panel indicates that fewer genes in the randomly selecting gene set are detected as members in Kyoto Encyclopedia of Genes and Genomes (KEGG) pathways than longevity‐associated genes (repeating 10 000 times). The median value of 10 000 values and the observed value based on longevity‐associated genes are presented using the red dotted lines. The right panel indicates a distribution of involved KEGG pathways (containing ≥15 longevity‐associated genes are presented here), and several pathways are the most dominant, including pathways in cancer, insulin signalling pathway, and mTOR‐signalling pathway. The distribution of the main involved KEGG pathways implicates the association of cancer and longevity. (F) Distributions of significantly enriched KEGG pathways and gene ontology (GO) terms based on the longevity‐associated genes (only the top enriched pathways or GO terms are presented here). Several longevity‐related pathways are highlighted in red, such as mTOR‐signalling pathway, longevity regulating pathway, and FoxO signalling pathway. The upper part is the distribution of the enriched KEGG pathway or GO term based on the gene count, and the lower part is based on the value of −log_10_ *p*_adj_. The dotted line shows *p*_adj_ value = .05. BP shows the biological process, CC shows the cellular component, and MF shows the molecular function. (G) The potential correlations of longevity‐associated genes and cancer‐related pathways across different cancers based on spearman correlation analysis. * indicates |cor| > .3 and FDR < .05. Freq indicates frequency, and cor indicates correlation coefficient. (H) Gene set variation analysis (GSVA) scores show the significant difference between tumour and normal samples in 14 cancers, and GSVA score in tumour samples (T) is always lower than that in normal samples (N) (*p* < 2.2e−16). The below panel shows that GSVA score is significantly lower in tumour samples than those in normal samples across different cancer types (* indicates *p* < .05).

**FIGURE 2:**
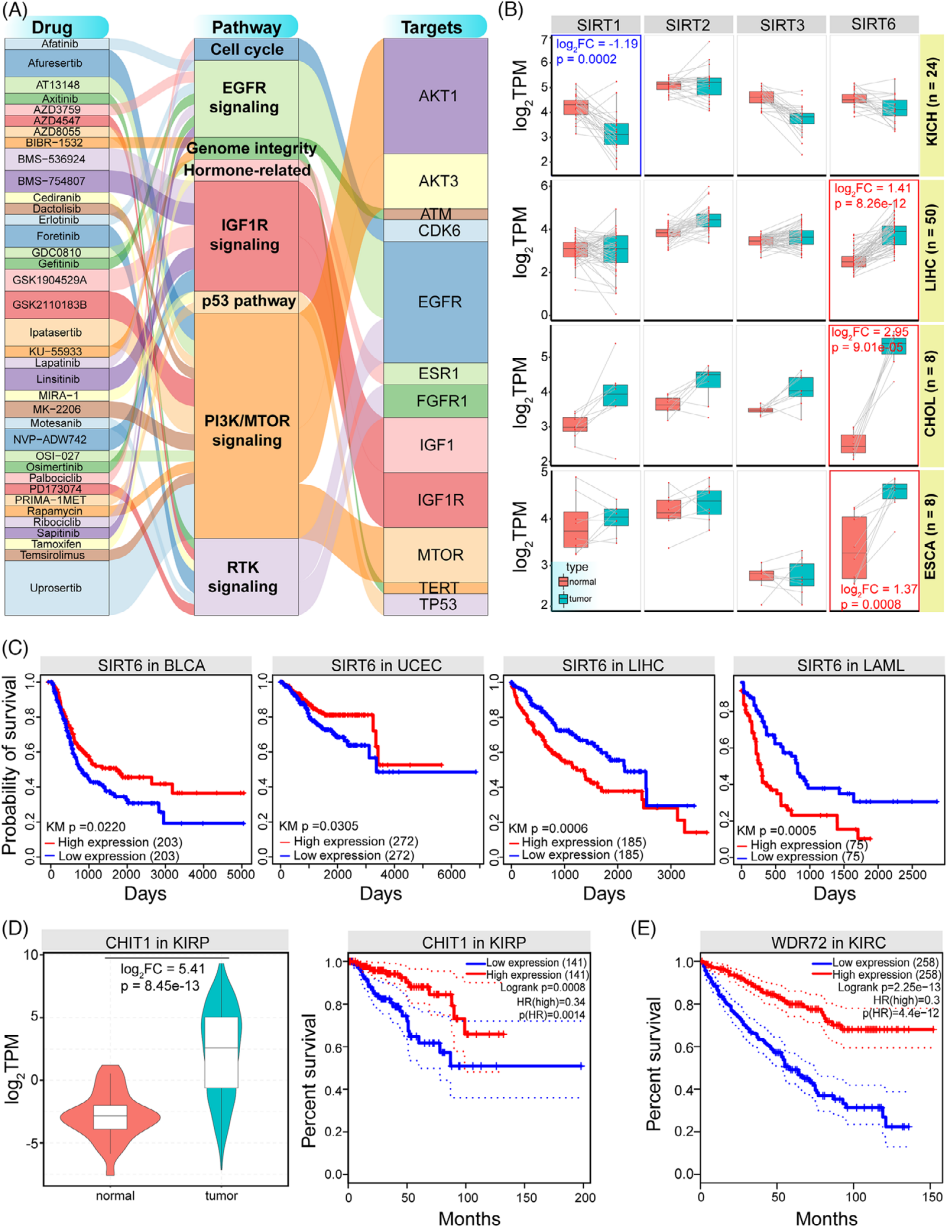
Expression profiles of longevity‐associated genes in pan‐cancer. (A) The collinearity graphs among drugs, signalling pathways and longevity‐associated genes are constructed using R “ggalliuvial” package, showing the roles of genes in relevant pathways as potential drug targets. Many genes are located in EGFR signalling and PI3K/MTOR signalling pathways, indicating that these genes may have critical roles in cancer treatment. The line thickness indicates the frequency of the corresponding relationship. (B) Examples of expression patterns of homologous members in the Sirtuin family in several cancer types based on the paired samples (sample sizes are also presented). Several homologous genes show the significant expression divergence in tumour samples than that in normal samples, but only SIRT6 is significantly up‐regulated in LIHC, esophageal carcinoma and cholangiocarcinoma, and a longevity‐related star gene, SIRT1, is significantly down‐regulated in KICH. SIRT6 may have a critical role in extending lifespan, and it may be a potential tumour suppressor to suppress cancer metabolism. (C) Based on the important role of SIRT6, the activity of the longevity‐associated gene may associate with outcome of cancers. Survival analysis shows that expression of SIRT6 may be associated with cancer prognosis in some cancer types (*p* < .05). Patients with higher expression of SIRT6 may have survival advantage than those with lower expression, especially for patients with BLCA and UCEC, while opposite result can be detected in LIHC and LAML. (D) An example of the expression distribution of CHIT1 in KIPR (CHIT1 is over‐expressed in tumour samples), and patients with high expression of CHIT1 shows the better survival advantage than patients with low expression. (E) Survival analysis of WDR72 in KIRC shows that patients with high expression indicate better survival than patients with low expression. BLCA, bladder urothelial carcinoma; EGFR, epidermal growth factor receptor.

To further validate the potential roles of longevity‐associated genes in classifying patients, consensus clustering analysis was conducted based on the expression level. Patients were divided into different groups that were associated with significant survival difference in some cancers (*p* < .05, Figures 3A and S5A). In liver hepatocellular carcinoma (LIHC), differential gene expression profiles between the two identified clusters mainly contributed to organic acid catabolic and metabolism‐related processes (Figure 3B,C). The two clusters also showed significantly different immune features (Figures 3D and S5B–E), implying the potential different immune‐related characteristics and immune microenvironment. Moreover, to estimate the potential prognostic value, multiple algorithms were used to screen critical genes (Figures 3E and S5F). The screened seven genes using the Enet algorithm were finally selected to construct a prognostic model for predicting survival, and the low‐risk group indicated the significant survival advantage than the high‐risk group (*p* < .0001, Figure 3F). Among these critical genes, CYP4F3 showed an important role in predicting survival probability (Figure 3G), followed by RDH16 that may be a potential prognostic biomarker for a novel therapeutic strategy in hepatocellular carcinoma,^9^ and it could be a pro‐metastasis biomarker.^10^ The prognostic model showed consistent results based on different datasets (Figure 3H), indicating a robust model. Some drugs, such as Alpelisib and Fulvestrant (Figure S5G), were sensitive to the high‐risk group, implicating a potential therapeutic effect. The prognostic ability in LIHC implicated the possible clinical application in the cancer treatment, and similar results could be detected in other cancer types, such as in uterine corpus endometrial carcinoma (UCEC) (Figure S6). These findings demonstrated that the longevity‐associated genes can be used to classify transcriptomic classes, which may be related with further cancer treatment.

**FIGURE 3:**
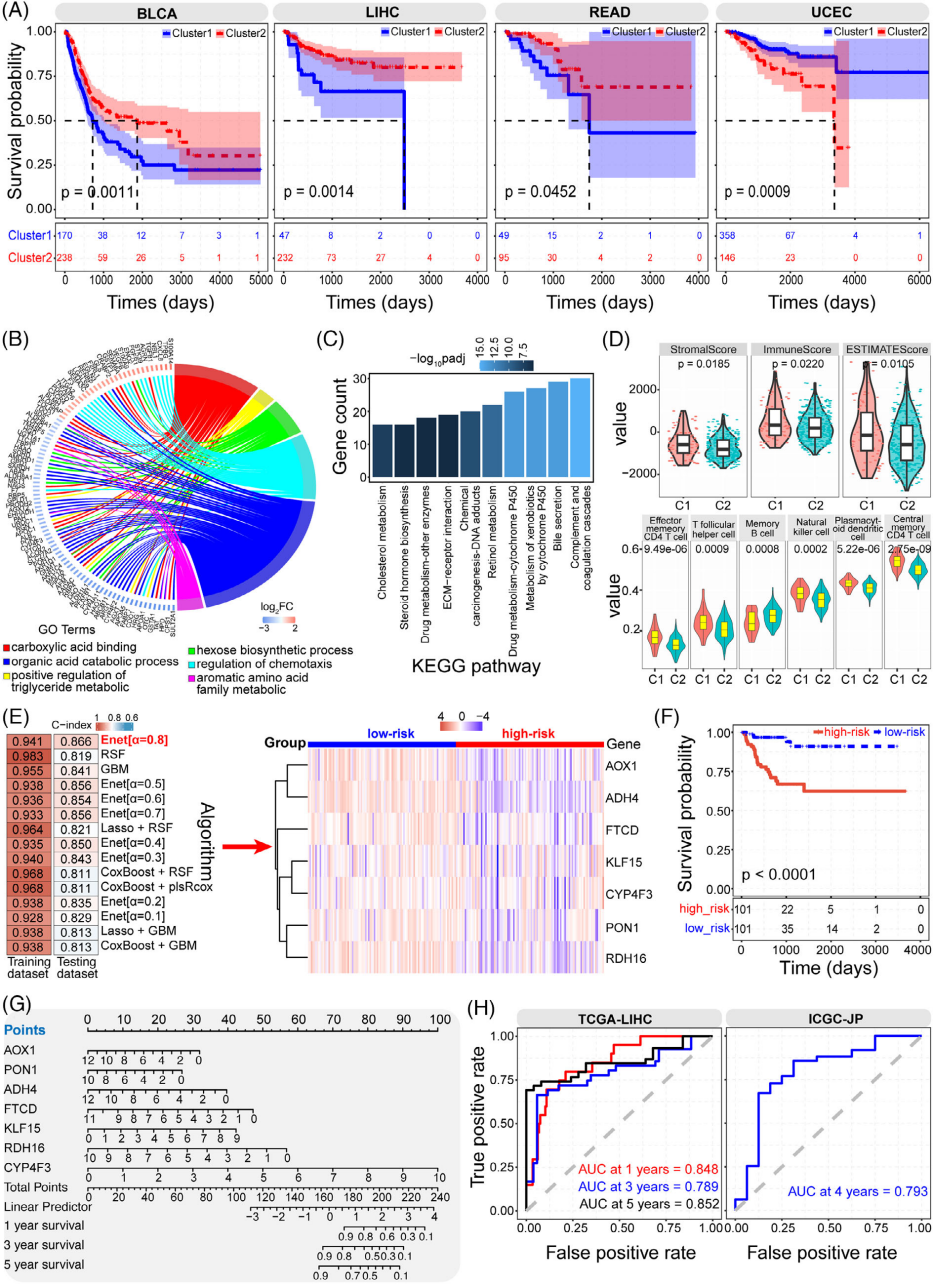
The potential role of longevity‐associated genes in cancer classifying. (A) There are two clusters classified based on the dysregulated expression of longevity‐associated genes in some cancer types using the non‐negative matrix factorisation (NMF) R package with the “brunet” standard, such as in BLCA, LIHC, READ and UCEC, and the two clusters show the significant survival difference (*p* < .05). (B,C) The enriched GO terms and KEGG pathways based on differentially expressed genes between the two classified groups in LIHC that is performed in‐depth analysis. These dysregulated genes between the two clusters mainly contribute to multiple biological processes and pathways, especially for organic acid catabolic process and metabolism‐related processes, indicating that patients in different clusters show the potential difference in metabolic processes. (D) The two clusters in LIHC show different immune‐related features (the above panel) and also show significant difference in some immune cells (the below panel). These results imply that the two constructed groups exist different immune‐related characteristics and immune microenvironment. (E) To estimate the potential prognostic value based on the two clusters in LIHC, multiple algorithms are used to screen candidate genes to construct the prognostic model. The top 15 C‐index distributions based on different combinations of 10 algorithms are presented (the left panel), and finally Enet (α = .8) is selected to screen seven candidate genes that can be used to construct the prognostic risk model (the right panel shows the detailed expression distributions of the seven screened genes between the high‐risk and the low‐risk groups). (F) The seven dysregulated genes in the two longevity‐associated clusters are used to construct a prognostic model, and the low‐risk group indicates the significant survival advantage than that in the high‐risk group (*p* < .0001). (G) Nomogram shows the contribution of different genes in the prognostic risk model. Among these dysregulated genes, CYP4F3 shows an important role in predicting survival probability, followed by RDH16. (H) The constructed prognostic model shows consistent results in different datasets based on the time‐dependent receiver operating characteristic curve, mainly including LIHC cohort in The Cancer Genome Atlas (TCGA‐LIHC cohort) and Japanese project from International Cancer Genome Consortium (ICGC‐JP cohort), implying that the model is robust in predicting the prognosis of LIHC patients. BLCA, bladder urothelial carcinoma.

Finally, to facilitate wider exploration of the longevity‐associated genes in cancer, we developed Longevity‐CancerDB (http://tmliang.cn/longene/) based on the screened longevity‐associated genes and relevant results, a user‐friendly web‐based resource, which can provide the detailed information and in‐depth pan‐cancer analysis to understand molecular changes, including levels of expression, mutation, methylation, copy number variation (CNV), survival, drug, etc. (Figure 4). In Longevity‐CancerDB, the longevity‐associated genes can be searched, downloaded, and analysed using online analysis tools to explore the detailed molecular features and potential clinical values in cancer.

**FIGURE 4:**
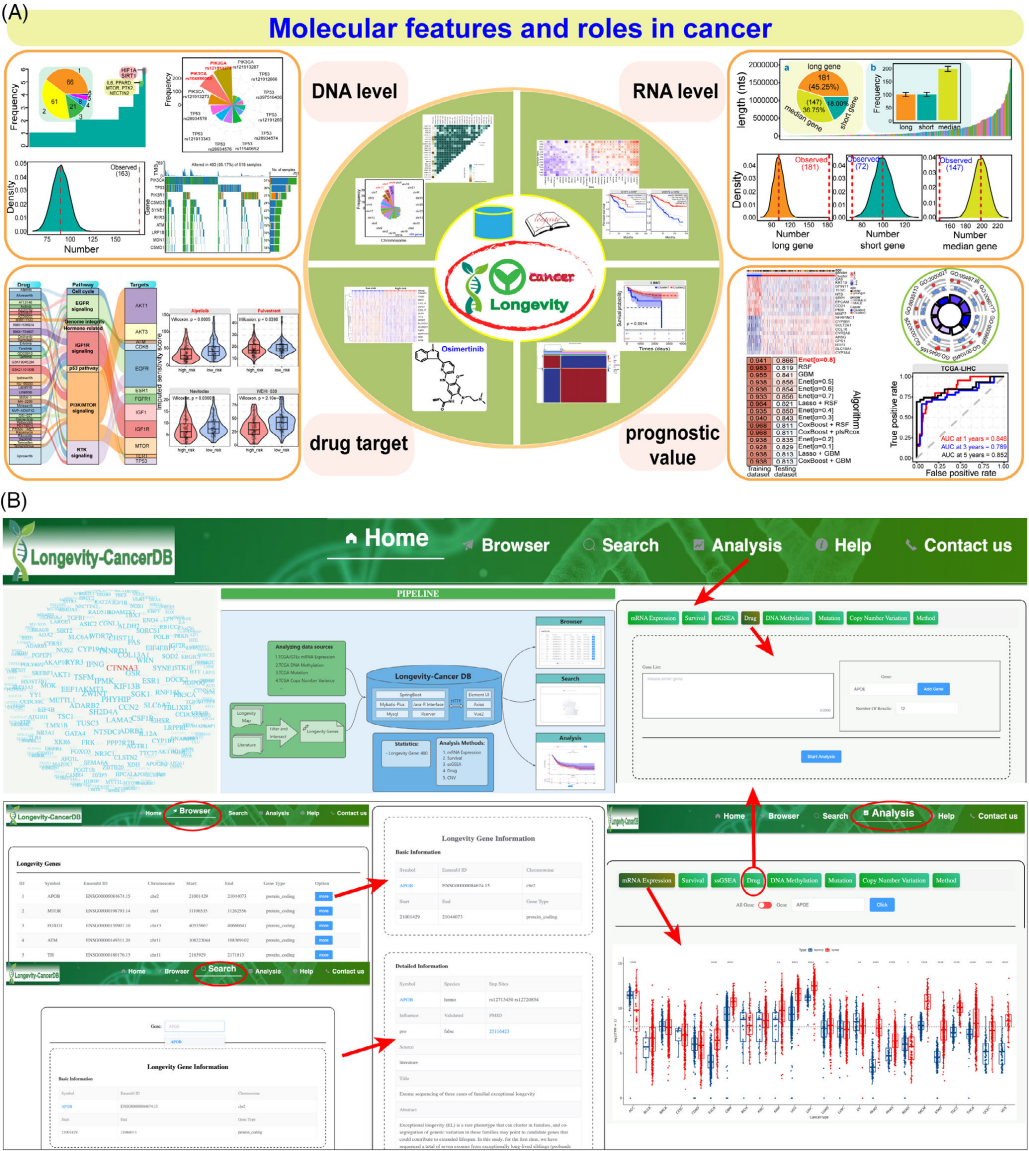
The main framework of pan‐analysis and the overview of longevity‐CancerDB. (A) A comprehensive analysis of longevity‐associated genes is performed via a multi‐omics approach to explore the potential molecular features and roles in cancer. (B) The main frame of developed Longevity‐Cancer Database based on the screened longevity‐associated genes and relevant results. Vue.js and Spring Boot framework are used to develop the website with back‐end separation. The online data analyses are visualised by Java calling R, the results are stored in MySQL, and the visual analysis and dynamic interaction of Longevity‐CancerDB are realised based on Rserve and echarts. In Longevity‐CancerDB, the screened longevity‐associated genes can be searched, downloaded and analysed using online analysis tools to understand the detailed molecular features and potential clinical values in cancer. This database provides the detailed information and in‐depth analysis for longevity‐associated genes, mainly including basic gene information and primary analysis of expression, mutation, methylation and copy number variation (CNV), and further analyses of survival, drug and single‐sample gene set enrichment analysis (ssGSEA) based on expression profiles are also provided.

Taken together, a systematic pan‐analysis of longevity‐associated genes was performed via a multi‐omics approach to explore the potential roles in cancer, and a user‐friendly Longevity‐CancerDB was then constructed to enrich the relevant studies (Figure 4). These genes tended to be cancer‐associated genes and may have pivotal roles in tumourigenesis via contributing multiple biological and cancer‐related pathways. Specific molecular features across different tissues can be detected, implying the potential contributions in tumourigenesis and cancer therapeutics, especially in personalised medicine for aging‐related diseases. According to these results, we further constructed Longevity‐CancerDB to enrich studies about the molecular characteristics and relationships between longevity and cancer. These findings can further broaden our understanding of longevity and cancer, which may highlight the potential roles and application of longevity‐associated genes in precision medicine.

## AUTHOR CONTRIBUTIONS

Li Guo and Tingming Liang participated in the conception and design of the study. Daoliang Xia, Jiaming Jin, Shizheng Xiong, Xinru Xu, Lulu Luo, Xueni Yang, Xinmiao Zhao, Dekang Ren and Jiafeng Yu carried out the experiments and analysed the data. Li Guo and Tingming Liang drafted the manuscript. Li Guo and Tingming Liang revised the manuscript. All authors have approved the final version of the manuscript.

## CONFLICT OF INTEREST STATEMENT

The authors declare no conflicts of interest.

## FUNDING INFORMATION

Supported by National Natural Science Foundation of China, Grant Number: 62171236; the key project of social development in Jiangsu Province, Grant Number: BE2022799; the key projects of Natural Science Research in Universities of Jiangsu Province, Grant Number: 22KJA180006; the Open Research Fund of State Key Laboratory of Bioelectronics, Southeast University,Grant Number: SKLB2022‐K03; funding from Shandong Provincial Key Laboratory of Biophysics, and the Priority Academic Program Development of Jiangsu Higher Education Institution (PAPD)

## ETHICS STATEMENT

All authors have been personally and actively involved in substantial work leading to the paper, and will take public responsibility for its content.

## Supporting information

Supporting Information

Supporting Information

Supporting Information

Supporting Information

Supporting Information

Supporting Information

Supporting Information

Supporting Information

Expression distributions of longevity‐associated genes in cancer.
Mutation distribution of some longevity‐associated genes.
An example of mutation landscape of longevity‐associated genes in UCEC.
The feature of gene length in longevity‐associated genes
The roles of longevity‐associated genes in LIHC classifying
The roles of longevity‐associated genes in UCEC classifying

## Data Availability

Longevity‐related genes were mainly obtained from publicly available database of LongevityMap (only significant associated gene were involved in further analysis) and experimentally validated longevity‐related genes from published literatures (Table S1). Sequencing data of mRNA, mutation, and relevant clinical data were collected from TCGA Web site using “TCGAbiolinks.” Simultaneously, other datasets were obtained for specific analysis, such as ICGC‐Japanese population (ICGC‐JP) used as the external validating dataset to verify the constructed prognostic model.
