# Supplementary figures and images for: Longevity‐CancerDB: unlocking the distinctive features and roles of longevity‐associated genes in tumourigenesis
Source: Clin Transl Med. 2024 Jan 22;14(1):e1557. doi: 10.1002/ctm2.1557 (PMC10802131; doi:10.1002/ctm2.1557)

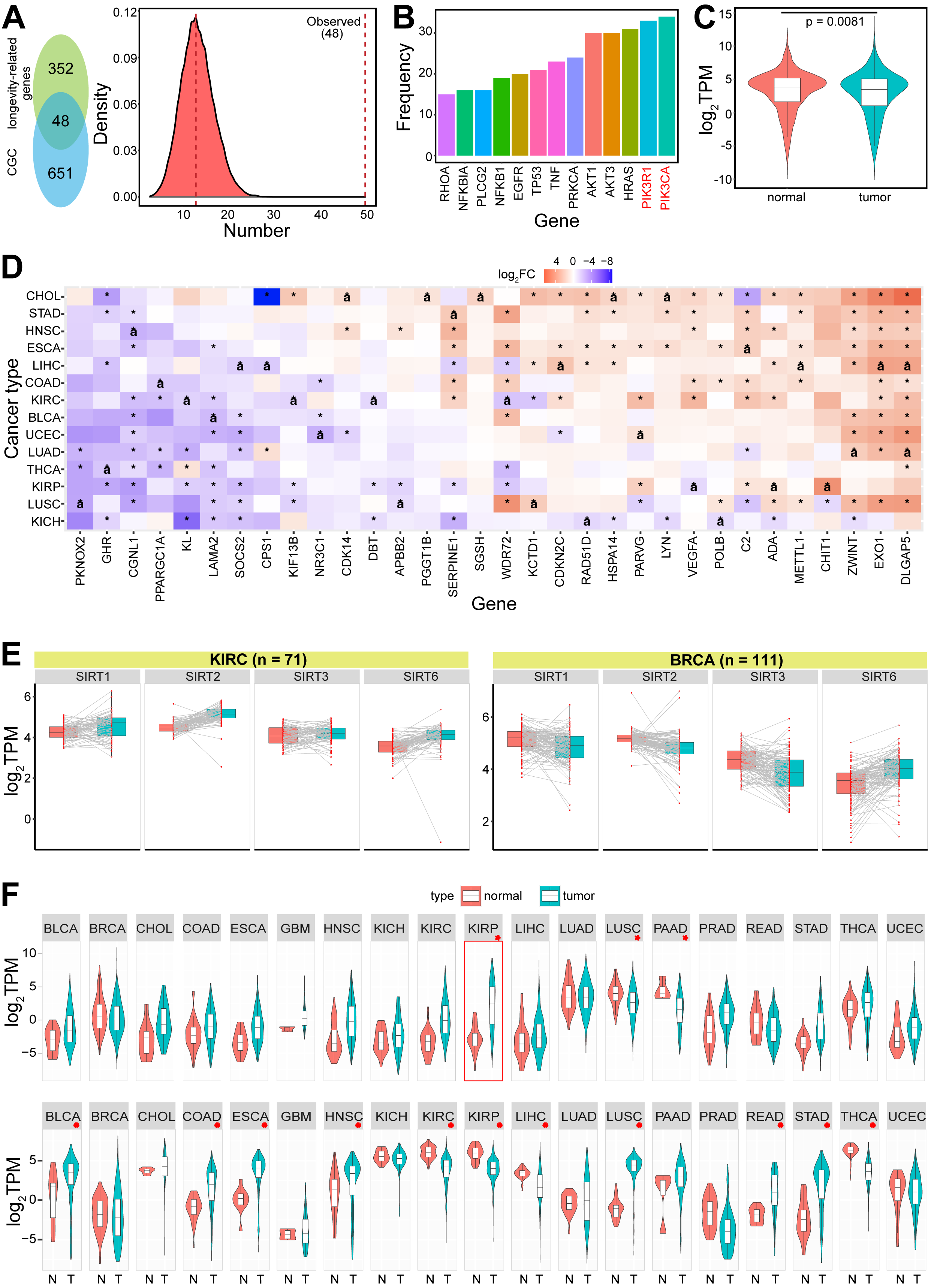

Supplement: Supplementary file 1 — Supporting Information [file CTM2-14-e1557-s003.tif]

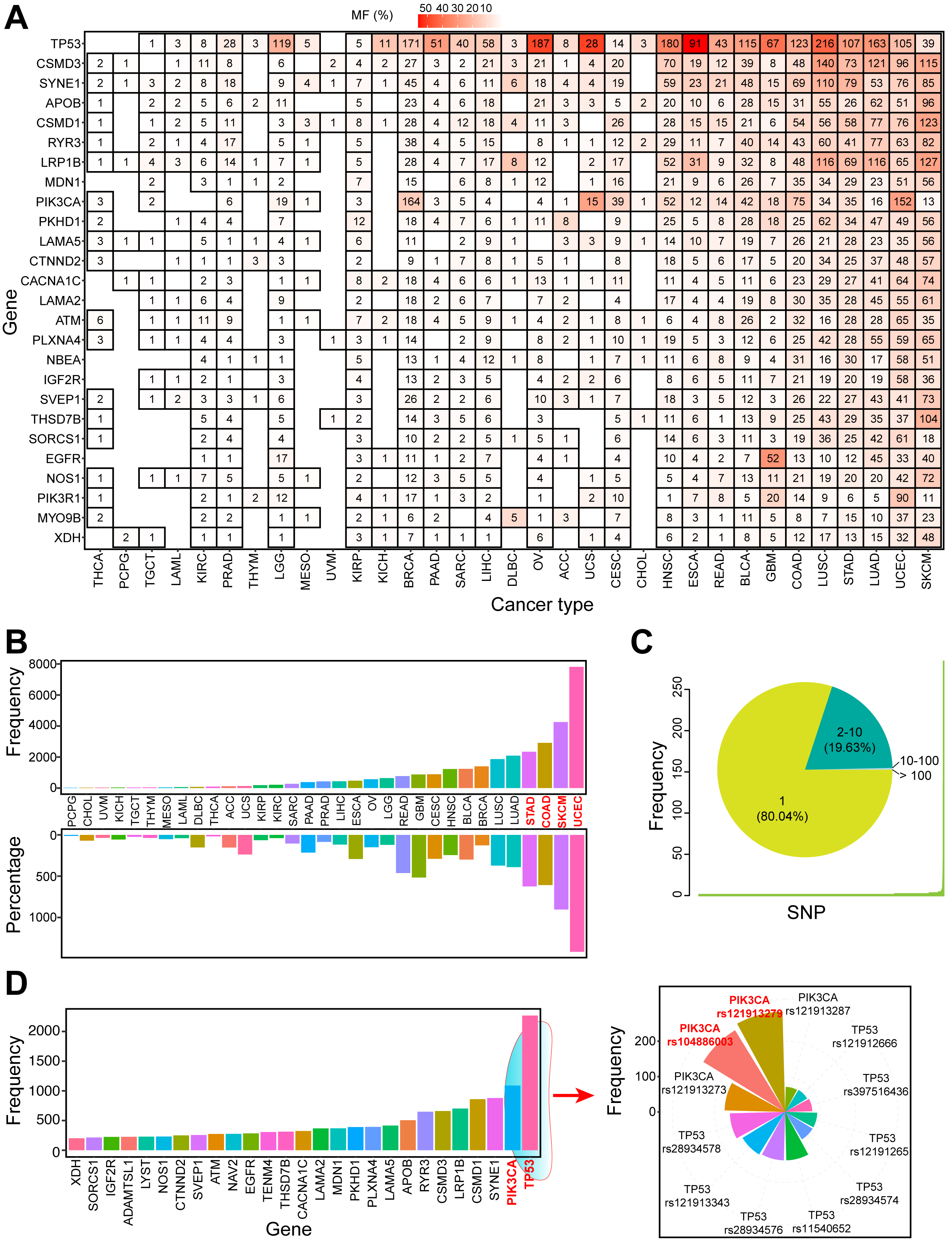

Supplement: Supplementary file 2 — Supporting Information [file CTM2-14-e1557-s002.tif]

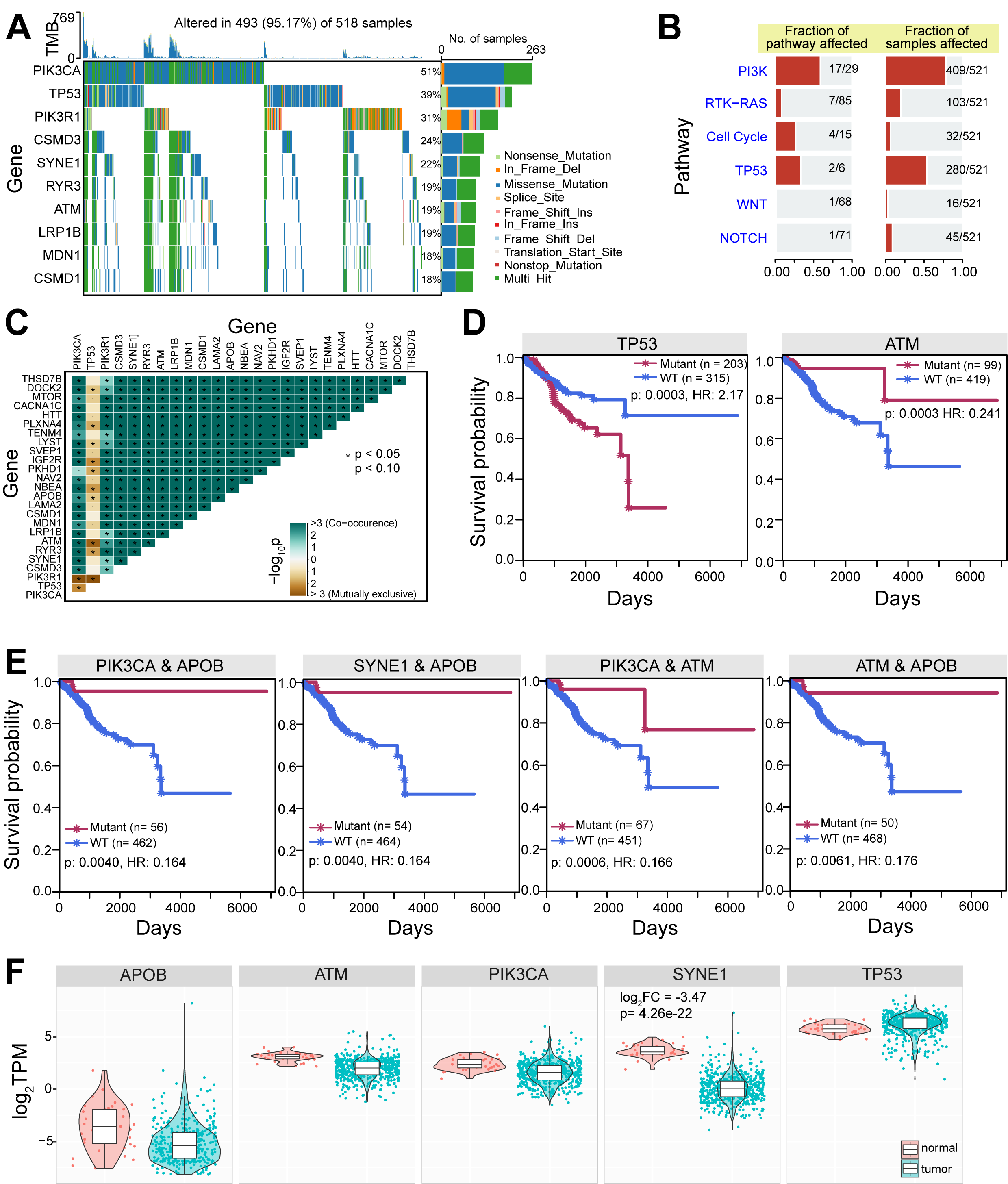

Supplement: Supplementary file 3 — Supporting Information [file CTM2-14-e1557-s009.tif]

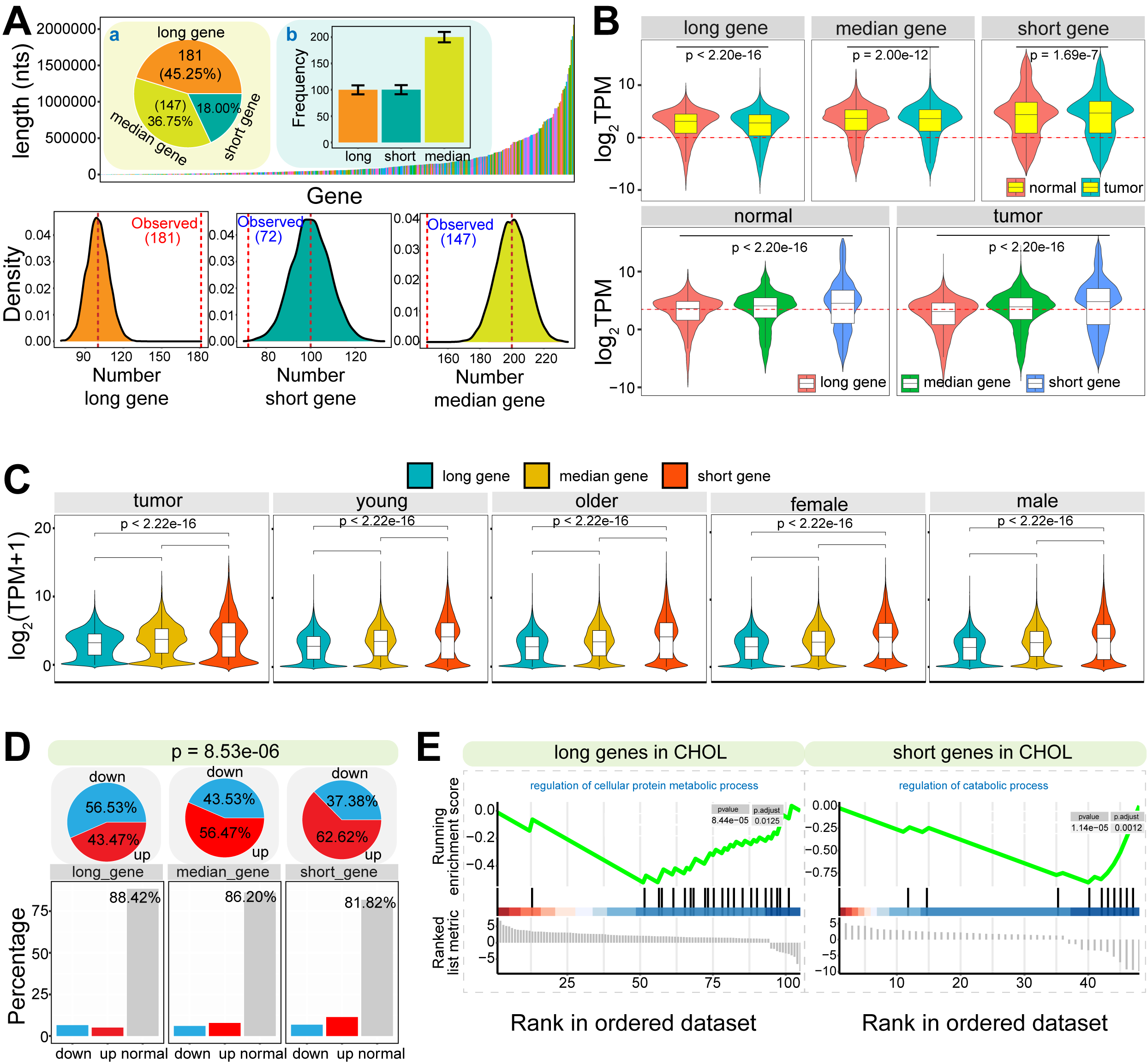

Supplement: Supplementary file 4 — Supporting Information [file CTM2-14-e1557-s006.tif]

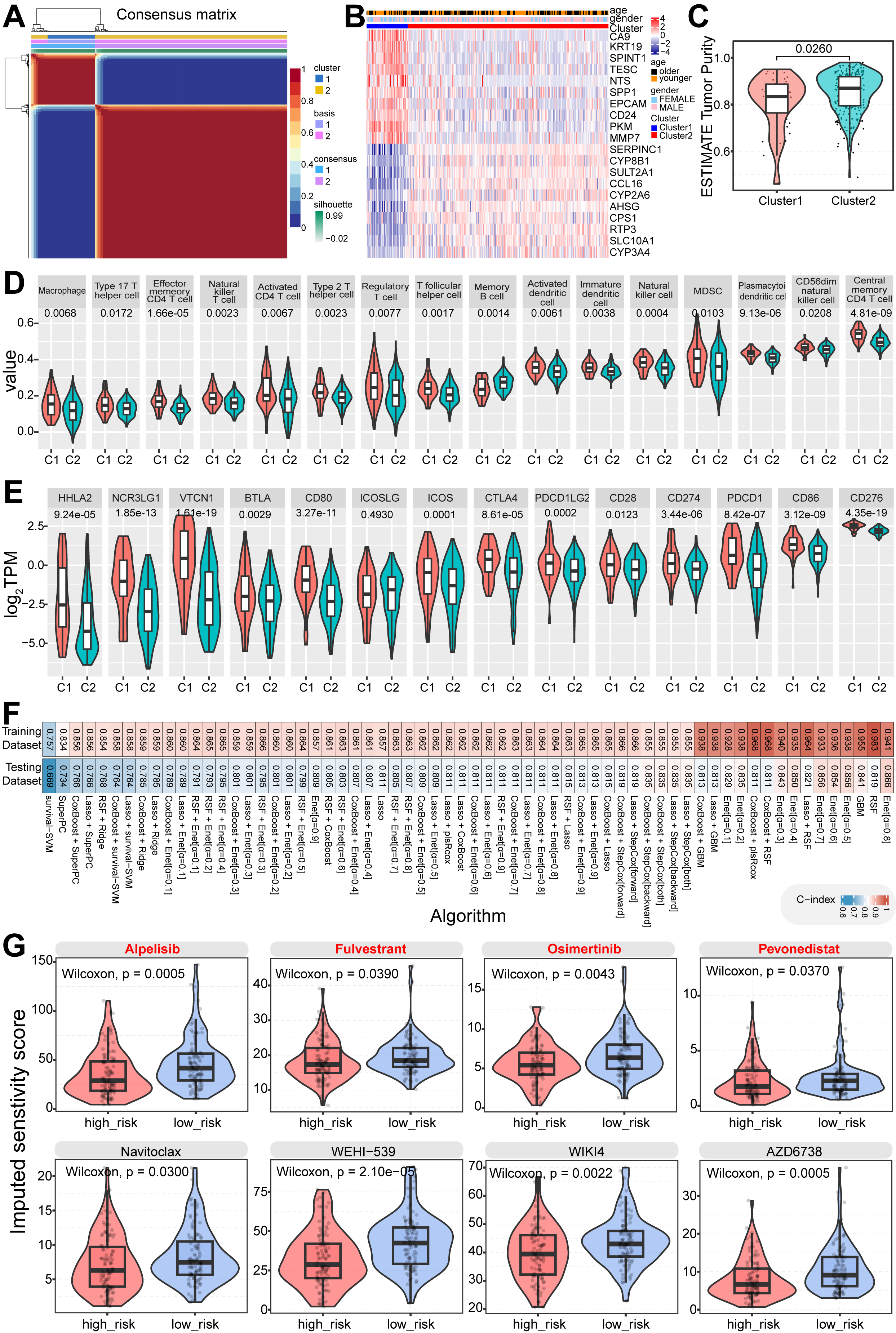

Supplement: Supplementary file 5 — Supporting Information [file CTM2-14-e1557-s008.tif]

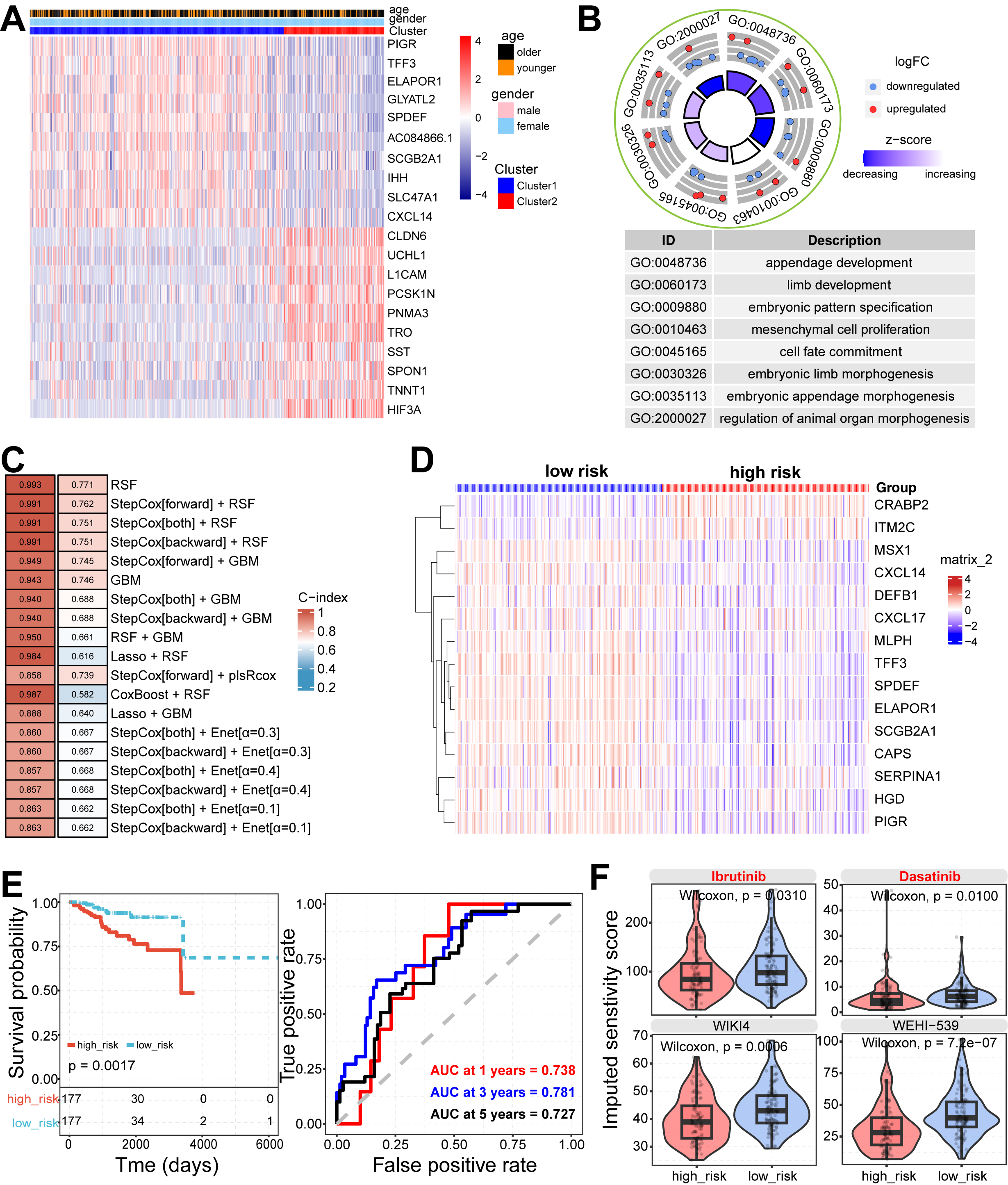

Supplement: Supplementary file 6 — Supporting Information [file CTM2-14-e1557-s007.tif]
