# Supplementary material for: Longevity‐CancerDB: unlocking the distinctive features and roles of longevity‐associated genes in tumourigenesis
Source: Clin Transl Med. 2024 Jan 22;14(1):e1557. doi: 10.1002/ctm2.1557 (PMC10802131; doi:10.1002/ctm2.1557)
Supplement: Supplementary file 8 — Supporting Information [file CTM2-14-e1557-s005.docx]

**Longevity-cancerDB: unlocking the distinctive features and roles of longevity-associated genes in tumorigenesis**

Li Guo^1^, Daoliang Xia^1^, Jiaming Jin^1^, Shizheng Xiong^1^, Xinru Xu^2^, Lulu Luo^2^, Xueni Yang^1^, Xinmiao Zhao^1^, Dekang Ren^1^, Jiafeng Yu^3^, Tingming Liang^2*^

**Running title:** Longevity-associated genes in cancer

^1^ State Key Laboratory of Organic Electronics and Information Displays, Institute of Advanced Materials (IAM), Nanjing University of Posts and Telecommunications, Nanjing 210023, China

^2^ Jiangsu Key Laboratory for Molecular and Medical Biotechnology, School of Life Science, Nanjing Normal University, Nanjing 210023, China

^3^ Shandong Provincial Key Laboratory of Biophysics, Institute of Biophysics, Dezhou University, Dezhou, 253023, China

*Corresponding author: Tingming Liang, Jiangsu Key Laboratory for Molecular and Medical Biotechnology, School of Life Science, Nanjing Normal University, Nanjing 210023, China

[Tel: +8602585891050](mailto:Tel:%20+8602585891050), Fax: +8602585891050, E-mail: [tmliang@njnu.edu.cn](mailto:tmliang@njnu.edu.cn)

**Materials and methods**

**Data resource**

Longevity-related genes were mainly obtained from publicly available database of LongevityMap [1] (only significant associated gene were involved in further analysis) and experimentally validated longevity-related genes from public references (Table S1). For these screened longevity-associated genes, to discuss the potential molecular features, sequencing data of mRNA, mutation and relevant clinical data, were collected from TCGA Web site using “TCGAbiolinks” [2]. Simultaneously, other datasets were obtained for specific analysis, such as ICGC-Japanese population (ICGC-JP) [3] were used as the external validating dataset to verify prognostic model.

**Differentially expressed RNA profiles and functional analysis**

The differential expression profiles for diverse RNAs were assessed using limma [4], and significantly deregulated genes were identified if |log_2_FC| > 1.2 and padj < 0.05. Functional analysis for involved genes was analyzed to understand the potential contributions in cancer pathophysiology using clusterProfiler 4.0 [5]. To understand the potential roles in tumorigenesis, relevant genes were further queried about contributions in hallmarks of cancer [6], Cancer Gene Census (CGC) [7, 8], oncogenes, and tumor suppressor genes (TSG) in OncoKB [9]. Simultaneously, based on the analysis results of longevity-related results, randomly selected 400 genes from all protein-coding genes (20,017 protein-coding genes according to gencode.v41.annotation.gtf) were performed analysis to assess whether the results of longevity-associated genes were random (repeating 10,000 times).

In addition, gene set variation analysis (GSVA) score for the longevity-related genes was simultaneously estimated in cancer using R package GSVA [10] and GSCA platform [11]. To understand the potential roles of longevity-associated genes in biological pathways and drug targets, the relations among genes, pathways and drugs were also calculated using oncoPredict R package [12] based on Genomics of Drug Sensitivity in Cancer (GDSC, https://www.cancerrxgene.org/) data [13].

**Analysis of somatic mutations**

To understand the somatic mutation profiles of longevity-associated genes in different cancers, genetic alterations were analyzed and visualized using “maftools” package (version 2.0.16)[14] based on the retrieved maf files for related cancer patients in the TCGA cohort from cBioPortal (http://cbioportal.org).

**Estimation of the potential roles of longevity-related genes in cancer prognosis**

In order to understand the potential roles of longevity-related genes in cancer prognosis, the Non-negative Matrix Factorization (NMF) R package with the “brunet” standard was used to identify the optimum number of clusters in pan-cancer based on dysregulated genes. The minimum member of each subtype was set to 10. The optimal number of clusters was then determined according to cophenetic, dispersion, and silhouette coefficients. For different subgroups, Kaplan-Meier survival analysis, functional enrichment analysis, immune-correlation analysis and drug sensitivity were performed to assess the potential difference of the two clusters. Further, spearman correlation analysis of immune cell subpopulations and sub-group was performed based on Estimate R package (https://bioinformatics.mdanderson.org/estimate/) to assess immune cell factors in different groups. Then, the cancer-infiltrating immune cells were scored via single-sample GSEA (ssGSEA) and the GSVA package [10] to quantify their relative contents. Simultaneously, the immune checkpoint activation between sub-groups was estimated via ggpubr R package (https://github.com/kassambara/ggpubr).

**Creation and validation of the prognosis risk assessment model**

To construct the prognosis risk assessment model, longevity-related genes associated with cancer prognosis between sub-groups were firstly screened using uni-COX regression (p < 0.05). Ten classical algorithms were integrated, including RSF, least absolute shrinkage and selection operator (LASSO), gradient boosting machine (GBM), survival support vector machine (Survival-SVM), supervised principal components (SuperPC), ridge regression, partial least squares regression for Cox (plsRcox), CoxBoost, Stepwise Cox, and elastic network (Enet). These machine-learning algorithm combinations were employed to select the most critical genes in cancer and subsequently to construct the risk model [15]. We then calculated the risk score using the signature obtained in the training cohort. After evaluating the average C-index of the testing cohorts, specific algorithms were ultimately selected and the best consensus prognosis model for cancers were then constructed. Finally, receiver operating characteristic (ROC) curve, area under the ROC curve (AUC) and the survival curve were used to validate the accuracy and prognostic value of the constructed model. Survival analysis was performed using Kaplan-Meier (KM) method to estimate the potential difference between different groups, and p value was calculated using the Log-rank test.

**Construction of a longevity-related model nomogram**

According to the clinical data and screened key genes in specific cancer types, an “rms” R package was used to draw a nomogram that could assess the different year overall survival (OS) of patients, and calibration curves to demonstrate the predictive power of the alignment chart.

**Statistical analysis and network visualization**

All statistical analyses were performed using R software (4.2.1). ROC curves and Kaplan-Meier log rank test in survival analysis were employed to predict the accuracy of the model. The independent predictive efficiency of the prognostic signature was assessed through univariate and multivariate cox analyses. The subgroups with different clinical characteristics were investigated to fully evaluate the stability of risk characteristics, and Student’s t-test and Wilcoxon signed-rank test were conducted to explore the differences between these subgroups. For paired samples, paired t-test was used to estimate the potential difference between tumor samples and adjacent normal samples. A p < 0.05 indicated statistically significant difference.

**Abbreviation lists in Figures**

ACC, adrenocortical carcinoma; BLCA, bladder urothelial carcinoma; BRCA, breast invasive carcinoma; CESC, Cervical squamous cell carcinoma and endocervical adenocarcinoma; CHOL, cholangiocarcinoma; COAD, colon adenocarcinoma; DLBC, lymphoid neoplasm diffuse large B-cell lymphoma; ESCA, esophageal carcinoma; GBM, glioblastoma multiforme; HNSC, head and neck squamous cell carcinoma; KICH, kidney chromophobe; KIRC, Kidney renal clear cell carcinoma; KIRP, kidney renal papillary cell carcinoma; LAML, acute myeloid leukemia; LIHC, liver hepatocellular carcinoma; LGG, brain Lower grade glioma; LUAD, lung adenocarcinoma; LUSC, lung squamous cell carcinoma; MESO, Mesothelioma; OV, ovarian serous cystadenocarcinoma; PAAD, pancreatic adenocarcinoma; PCPG, pheochromocytoma and paraganglioma; PRAD, prostate adenocarcinoma; READ, rectum adenocarcinoma; SARC, sarcoma; SKCM, skin cutaneous melanoma; STAD, stomach adenocarcinoma; TGCT, testicular germ cell tumors; THCA, thyroid carcinoma; THYM, thymoma; UCEC, uterine corpus endometrial carcinoma; UCS, uterine carcinosarcoma; UVM, uveal melanoma.

**References**

[1] Budovsky A, Craig T, Wang J, Tacutu R, Csordas A, Lourenco J, et al. LongevityMap: a database of human genetic variants associated with longevity. Trends Genet. 2013;29:559-60.

[2] Colaprico A, Silva TC, Olsen C, Garofano L, Cava C, Garolini D, et al. TCGAbiolinks: an R/Bioconductor package for integrative analysis of TCGA data. Nucleic Acids Res. 2016;44:e71.

[3] Fujimoto A, Furuta M, Totoki Y, Tsunoda T, Kato M, Shiraishi Y, et al. Whole-genome mutational landscape and characterization of noncoding and structural mutations in liver cancer. Nat Genet. 2016;48:500-9.

[4] Ritchie ME, Phipson B, Wu D, Hu Y, Law CW, Shi W, et al. limma powers differential expression analyses for RNA-sequencing and microarray studies. Nucleic Acids Res. 2015;43:e47.

[5] Wu T, Hu E, Xu S, Chen M, Guo P, Dai Z, et al. clusterProfiler 4.0: A universal enrichment tool for interpreting omics data. Innovation (Camb). 2021;2:100141.

[6] Hanahan D, Weinberg RA. Hallmarks of cancer: the next generation. Cell. 2011;144:646-74.

[7] Futreal PA, Coin L, Marshall M, Down T, Hubbard T, Wooster R, et al. A census of human cancer genes. Nat Rev Cancer. 2004;4:177-83.

[8] Sondka Z, Bamford S, Cole CG, Ward SA, Dunham I, Forbes SA. The COSMIC Cancer Gene Census: describing genetic dysfunction across all human cancers. Nat Rev Cancer. 2018;18:696-705.

[9] Chakravarty D, Gao J, Phillips SM, Kundra R, Zhang H, Wang J, et al. OncoKB: A Precision Oncology Knowledge Base. JCO Precis Oncol. 2017;2017.

[10] Hanzelmann S, Castelo R, Guinney J. GSVA: gene set variation analysis for microarray and RNA-seq data. BMC Bioinformatics. 2013;14:7.

[11] Liu CJ, Hu FF, Xia MX, Han L, Zhang Q, Guo AY. GSCALite: a web server for gene set cancer analysis. Bioinformatics. 2018;34:3771-2.

[12] Maeser D, Gruener RF, Huang RS. oncoPredict: an R package for predicting in vivo or cancer patient drug response and biomarkers from cell line screening data. Brief Bioinform. 2021;22.

[13] Yang W, Soares J, Greninger P, Edelman EJ, Lightfoot H, Forbes S, et al. Genomics of Drug Sensitivity in Cancer (GDSC): a resource for therapeutic biomarker discovery in cancer cells. Nucleic Acids Res. 2013;41:D955-61.

[14] Mayakonda A, Lin DC, Assenov Y, Plass C, Koeffler HP. Maftools: efficient and comprehensive analysis of somatic variants in cancer. Genome Res. 2018;28:1747-56.

[15] Liu Z, Liu L, Weng S, Guo C, Dang Q, Xu H, et al. Machine learning-based integration develops an immune-derived lncRNA signature for improving outcomes in colorectal cancer. Nat Commun. 2022;13:816.
