# Supplementary material for: Longevity‐CancerDB: unlocking the distinctive features and roles of longevity‐associated genes in tumourigenesis
Source: Clin Transl Med. 2024 Jan 22;14(1):e1557. doi: 10.1002/ctm2.1557 (PMC10802131; doi:10.1002/ctm2.1557)
Supplement: Supplementary file 9 — Expression distributions of longevity‐associated genes in cancer. Mutation distribution of some longevity‐associated genes. An example of mutation landscape of longevity‐associated genes in UCEC. The feature of gene length in longevity‐associated genes The roles of longevity‐associated genes in LIHC classifying The roles of longevity‐associated genes in UCEC classifying [file CTM2-14-e1557-s001.docx]

**Supplementary files**

**Figure S1. Expression distributions of longevity-associated genes in cancer.**

A. The common 48 genes (12% of longevity-associated genes) can be detected between Cancer Gene Census (CGC) and longevity-associated genes. The right panel indicates that fewer genes in the randomly selecting gene set (n = 400) are detected as CGC than longevity-associated genes (repeating 10,000 times). The left red dotted line shows the median value of 10,000 values, and the red dotted line shows the observed value based on longevity-associated genes.

B. The distribution of some genes involved in KEGG pathways. Many genes are identified as members in multiple KEGG pathways, especially for PIK3CA and PIK3R1.

C. Significant expression difference (p = 0.0081 using Wilcoxon signed-rank test) can be detected between all tumor and normal samples based on the gene set of longevity-associated genes.

D. An example of expression patterns of dysregulated longevity-associated genes in pan-cancer. * indicates significantly dysregulated dominantly expressed genes (AveExpr value > 0, |log_2_FC| > 1.2 and padj < 0.05 based on limma), and a indicates that this gene shows significant survival difference between high and low expression in specific cancer type.

E. Stable expression patterns of homologous members can be found in Sirtuin gene family in some cancer types based on paired tumor and normal samples.

F. Expression analysis of CHIT1 in pan-cancer. * indicates that CHIT1 is significantly dysregulated.

G. Expression analysis of WDR72 in pan-cancer indicates diverse expression patterns in different cancers. * indicates that WDR72 is significantly dysregulated (|log_2_FC| > 1.2 and padj < 0.05). N indicates normal samples, and T indicates tumor samples.

**Figure S2. Mutation distribution of some longevity-associated genes.**

A. The mutation distributions in pan-cancer based on the top 26 longevity-associated genes with higher mutation frequencies.

B. The distribution of mutation frequency based on all single nucleotide polymorphisms (SNP) mutations across cancer types (the up panel), and the distribution of relevant percentage is also presented based on the total sample size of tumor samples (the low panel).

C. The distribution of the detailed mutation and pie distribution show most mutations are only detected in specific patient (more than 80%).

D. The distribution of the top 27 genes with higher mutation frequencies based on SNP mutation in all samples. TP53 and PIK3CA are the top two genes with higher mutation frequencies, and the frequencies of several SNP mutations are also presented, especially for rs121913279 and rs104886003 of PIK3CA.

**Figure S3. An example of mutation landscape of longevity-associated genes in UCEC.**

A. The mutation profiles of the top 10 genes with higher mutation frequencies in UCEC that is taken an example to present mutation landscape. The somatic mutation profiles in different cancers and genetic alterations are analyzed and visualized using “maftools” package (version 2.0.16) based on the retrieved maf files for related cancer patients in the TCGA cohort from cBioPortal (http://cbioportal.org).

B. The dominant pathways based on the top 20 genes with higher mutation frequencies, mainly including PI3K, TP53 and cell cycle pathways.

C. The correlations of the top 20 genes show that most gene combinations are co-occurrence patterns, but TP53 and PIK3CA show a mutually exclusive pattern.

D. Examples of survival analysis indicate the potential prognostic values of some longevity-associated genes, such as TP53 and ATM. The wildtype group of TP53 shows better survival than that in the mutant group, but the mutant group of ATM shows better survival than that in the wildtype group.

E. Examples of survival analysis of gene combination based on the top 20 genes with higher mutation frequencies, and some paired genes are associated with cancer prognosis.

F. Expression distributions of involved genes in Figure S3E. APOB is detected with lower expression levels. All of these genes are differentially expressed between tumor and normal samples (p < 0.05), but SYNE1 is significantly down-regulated in tumor samples (log_2_FC = -3.47, p = 4.26e-22 based on Wilcoxon test).

**Figure S4. The feature of gene length in longevity-associated genes.**

A. Based on the potential function of long genes in tumorigenesis, these longevity-associated genes are divided into long genes (genes with more than 71,733 nucleotides are defined as long genes, the top 25.00% of all protein-coding genes according to the gene length), short genes (< 9,717 nucleotides, the last 25.00% of all protein-coding genes according to the gene length), and others are median genes. The up panel is distribution of gene length based on 400 longevity-associated genes. a indicates the number distribution of long, median and short genes, and long genes are the most dominant (possess 45.25%). b indicates the average distributions of genes with different lengths (mean ± standard deviation) based on randomly sampling from all protein-coding genes (n = 400, repeating 10,000 times), and the median genes are the most dominant based on random sampling. The down panel is the detailed distributions based on randomly selecting genes from all protein-coding genes. The average number based on random sampling and the observed gene numbers in longevity-associated genes are also highlighted. All of these results indicate that longevity-associated genes tend to be long genes.

B. The up panel indicates the expression distributions of different genes between all normal and tumor samples in pan-cancer. The p values based on Wilcoxon test are presented. The down panel indicates the detailed expression distributions of different genes in breast cancer because of the larger sample size than other cancer types. Short genes are prone to show abundant expression patterns than long and median genes.

C. The expression distributions of genes with diverged length between different populations, including all tumor samples in pan-cancer, young samples (<= 65), older samples (> 65), female and male samples. The consistent expression patterns can be detected, short genes show higher expression levels, while long genes indicate lower expression levels than other gene types.

D. The detailed expression pattern of different genes in pan-cancer. A p value based on Pearson's Chi-squared test has been presented for distributions of up-regulated and down-regulated genes. Genes are defined as dysregulated genes if AveExpr > 2, |log_2_FC| > 1.2 & padj < 0.05 in specific cancer type.

E. An example of Gene Set Enrichment Analysis (GSEA) analysis of long genes and short genes in CHOL, showing that the long genes and short genes are specifically enriched in regulation of cellular protein metabolic process and regulation of catabolic process, respectively.

**Figure S5. The roles of longevity-associated genes in LIHC classifying.**

A. A heatmap shows consensus matrix for k = 2 obtained by applying NMF.

B. The two clusters (C1 and C2) show distinct features based on the top 20 genes.

C. The two clusters show significantly different estimate tumor purity (p = 0.0260).

D. The two clusters show significant difference in some immune cells.

E. Some checkpoint genes show different expression patterns between the two clusters.

F. The C-index distributions based on different combinations of 10 algorithms and finally Enet (α = 0.8) is selected.

G. Examples of sensitivities of some drugs are significantly different between the two groups (high risk and low risk groups) in LIHC, such as Alpelisib, Fulvestrant, Osimertinib and Pevonedistat, and these drugs are sensitive in the high-risk group than those in the low-risk group.

**Figure S6. The roles of longevity-associated genes in UCEC classifying.**

A. The detailed expression patterns of the top 20 dysregulated genes in the constructed two clusters of UCEC based on distinct features using NMF.

B. The enriched GO terms based on differentially expressed genes between the two classified groups in UCEC.

C. The C-index distributions based on different combinations of 10 algorithms (some results are presented here) and finally random survival forests (RSF) algorithm is selected.

D. The expression distributions of the screened genes in high and low risk groups using RSF algorithm, indicating that the high risk and low risk groups have significant different expression patterns.

E. The overall survival (OS) indicates significant survival difference between the high-risk and low-risk groups in UECE (p = 0.0017, the left panel). The right panel shows that the prognostic accuracies are 0.738 at 1 years, 0.781 at 3 years and 0.727 at 5 years based on the TCGA-UCEC cohort.

F. Examples of sensitivities of some drugs are significantly different between the two groups (high risk and low risk groups) in UCEC.

**Table S1. Screened 400 longevity-associated genes in this study.**
